# Supplementary material for: StrataChip: a microphysiological system to capture dynamic keratinocyte fate and mechanical transitions during human epidermal morphogenesis
Source: NPJ Biomed Innov. 2026 Nov 12;3:68. doi: 10.1038/s44385-026-00124-3 (PMC13759492; doi:10.1038/s44385-026-00124-3)

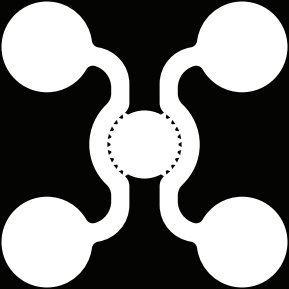

Supplement: Supplementary file 1 [file 44385_2026_124_MOESM1_ESM.pdf]
