# Supplementary material for: StrataChip: a microphysiological system to capture dynamic keratinocyte fate and mechanical transitions during human epidermal morphogenesis
Source: NPJ Biomed Innov. 2026 Nov 12;3:68. doi: 10.1038/s44385-026-00124-3 (PMC13759492; doi:10.1038/s44385-026-00124-3)
Supplement: Supplementary file 2 [file 44385_2026_124_MOESM2_ESM.pdf]

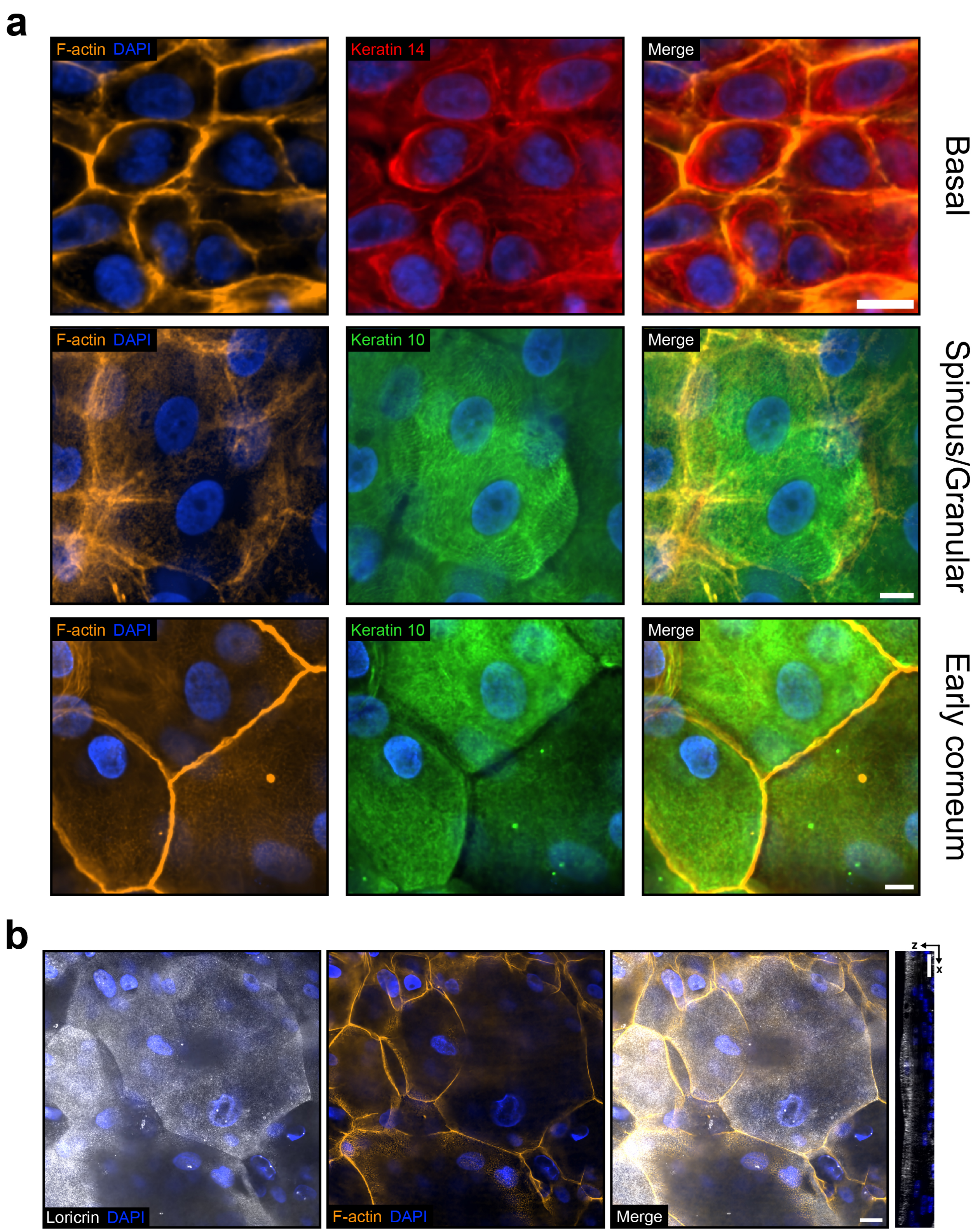

Supplementary Figure 1: Keratin filaments in different layers of model epidermis

(a) immunofluorescence X-Y micrographs of keratin filaments in the basal, spinous/granular, and corneum layers of the model epidermis. **(b)** immunofluorescence micrographs of loricrin in outer most of epidermis.

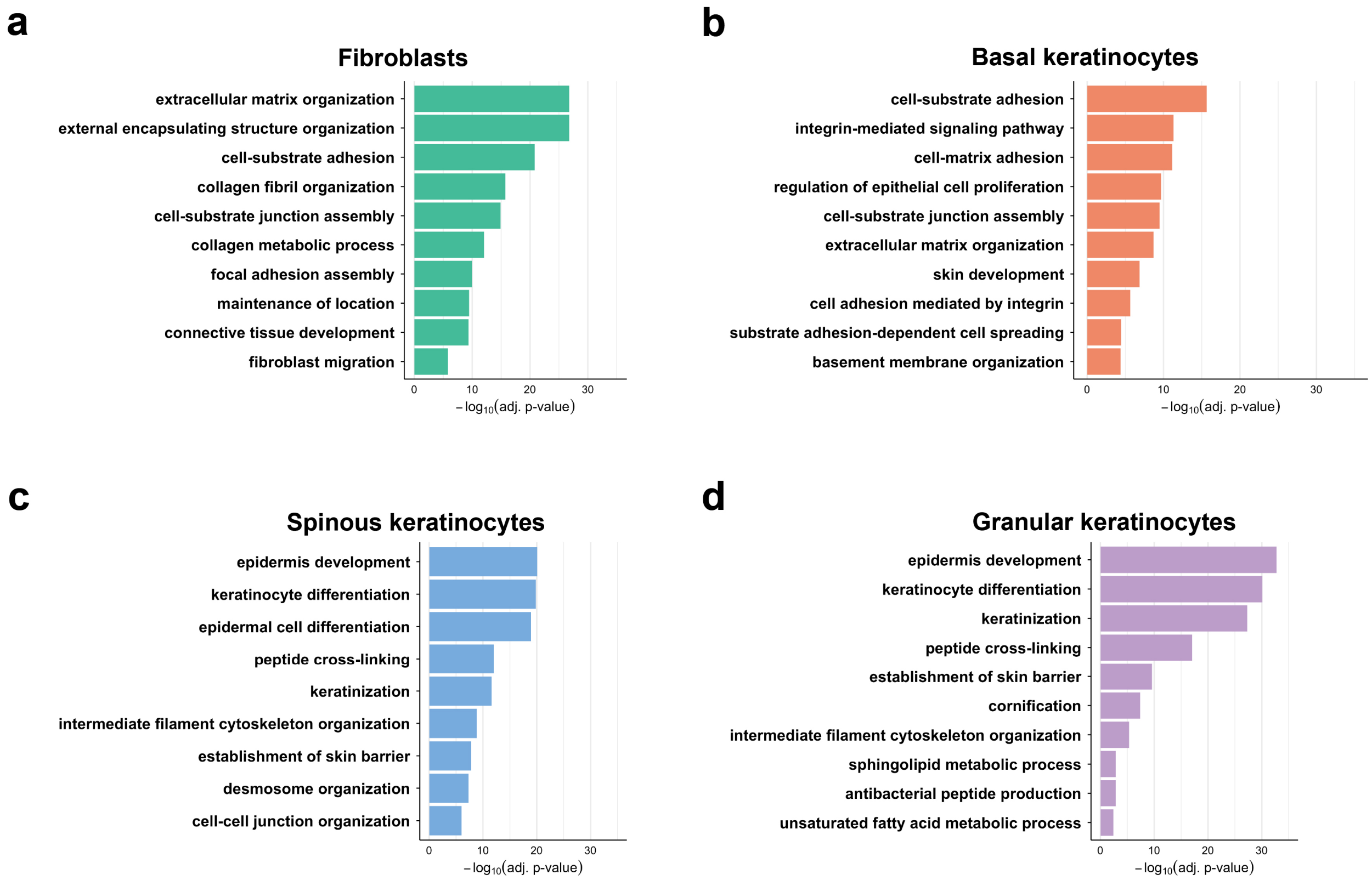

Supplementary Figure 2: Top ten relevant GO terms from fibroblasts and each keratinocyte fate.

(a) - (d) Top ten relevant Gene Ontology (GO) terms from the cell fates: fibroblasts and basal, spinous, and granular keratinocytes.

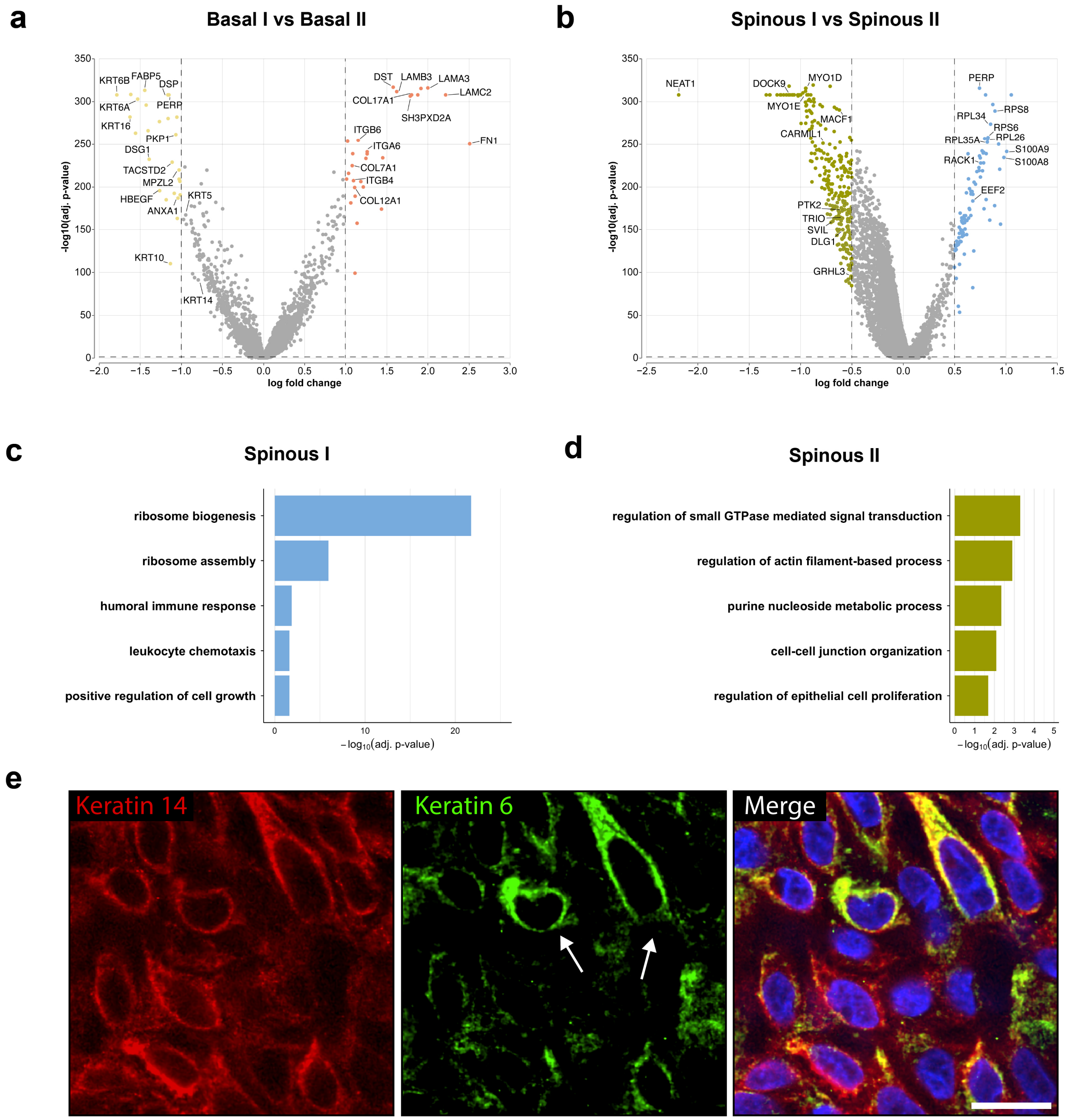

Supplementary Figure 3: Transcriptomic differences within keratinocyte cell fates.

(**a**) Differentially expressed genes between basal I versus basal II keratinocytes. **(b)** Differentially expressed genes between spinous I versus spinous II keratinocytes. **(c) and (d)** Enriched GO terms of spinous I and spinous II keratinocytes when compared with each other. **(e)** immunofluorescence micrographs of keratin 14 and keratin 6 in basal layer of model epidermis. Arrows point to high keratin 6 positive basal keratinocytes.

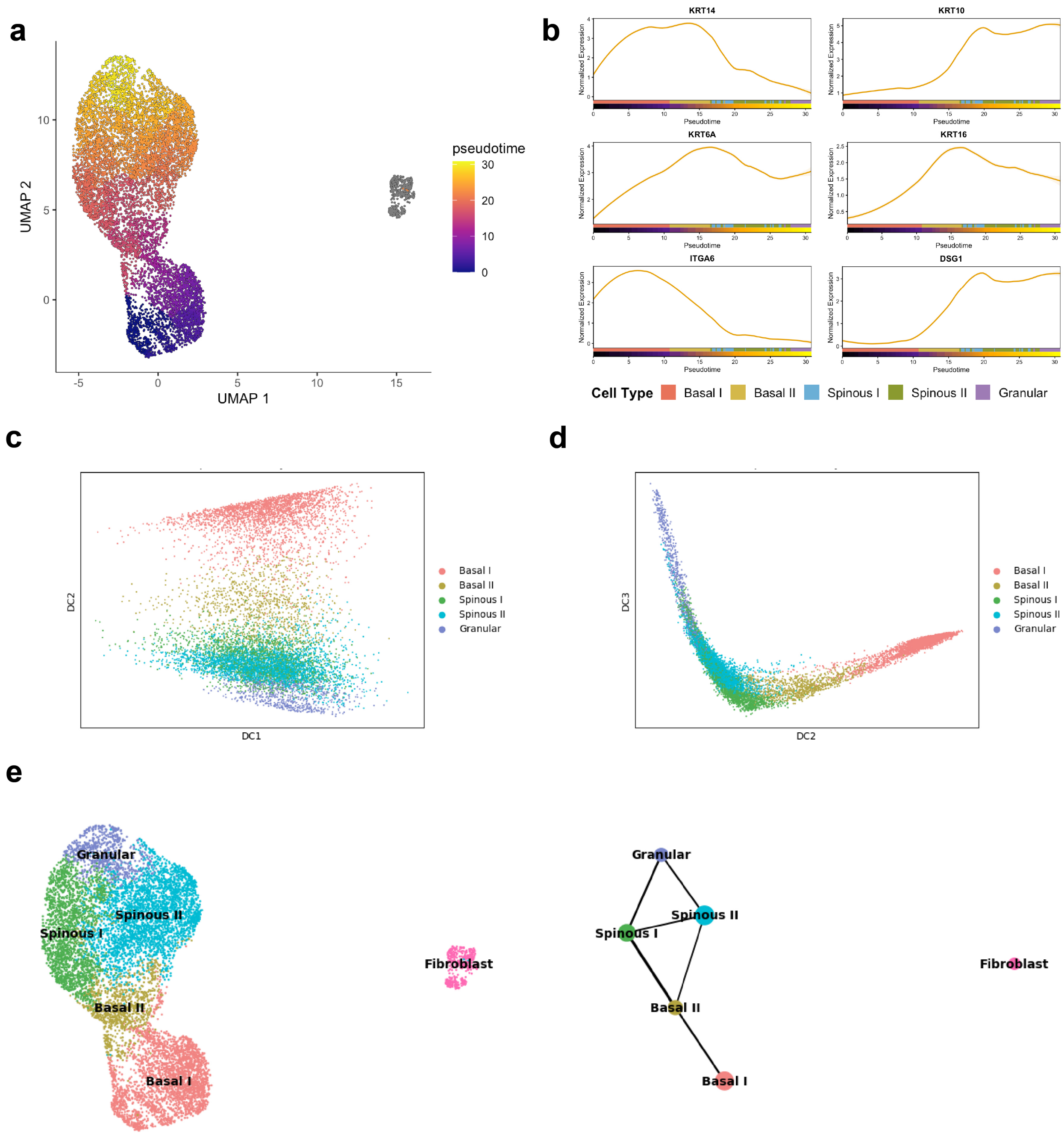

Supplementary Figure 4: Lineage relationships in StrataChip model epidermis

(a) Monocle3 pseudotime UMAP of cell clusters from StrataChip. **(b)** Pseudotime plot of various keratin and adhesion molecules transcripts. **(c)-(d)** Diffusion maps of StrataChip keratinocyte subpopulations. **(e)** Partition-based graph abstraction (PAGA) analysis of StrataChip keratinocyte subpopulations.

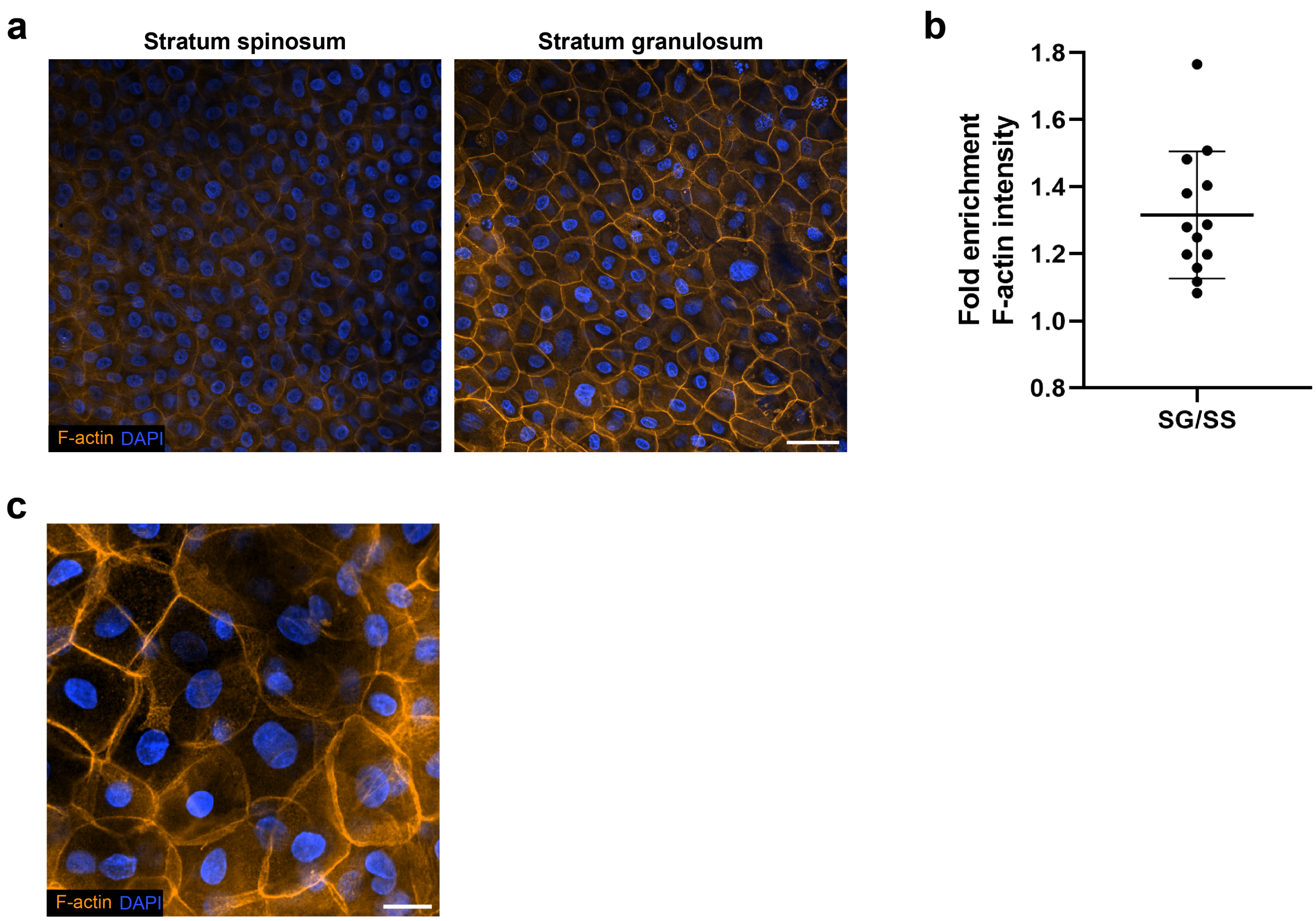

Supplementary Figure 5: StrataChip captures keratinocyte heterogeneity and asymmetry.

(a) Fluorescence micrographs of epidermal spinous layer and granular layer labeled for F-actin and DAPI. Scale bar 50 μm. **(b)** Normalized fold enrichment of F-actin intensity in granular layer of StrataChip epidermis. Stratum spinosum (SS) and stratum granulosum (SG). N = 13. **(c)** Fluorescence micrograph showing heterogenous F-actin intensity in granular layer of epidermis labeled with F-actin and DAPI. Scale bar 20 μm.

Supplementary Movie 1 - Transition through the StrataChip epidermis in z. F-actin in black and nuclei in blue. Z-step, 0.4 µm and z-depth, 49.2 µm. Displayed at 20 frames/z-steps per second. Scale bar, 50 µm.

Supplementary Movie 2 - Spy555-FastAct (white) and Spy650-DNA (blue) labeled keratinocytes in StrataChip visualized via time-lapse light sheet microscopy. Frames collected every 3 mins and displayed at 7 frames per second. Z-step, 0.23 nm/pixel. Scale bar, 20 µm.

Supplementary Movie 3 - Figure 4 delamination event. Spy555-FastAct (white) labeled keratinocytes in StrataChip visualized via time-lapse confocal microscopy. Frames collected every 5 mins and displayed at 3 frames per second. Z-step, 1 µm. Scale bar, 20 µm.

Supplementary Movie 4 - Figure 4 Imaris delamination event reconstruction. Displayed at 3 frames per second. Scale bar, 10 µm.

Supplementary Movie 5 - Figure 4 Imaris delamination event reconstruction bottom POV. displayed at 3 frames per second. Scale bar, 10 µm.

Supplementary Movie 6 - Figure 4 asymmetric division event. Spy555-FastAct (white) labeled keratinocytes in StrataChip visualized via time-lapse confocal microscopy. Frames collected every 5 mins and displayed at 3 frames per second. Z-step, 1 µm. Scale bar, 20 µm.

Supplementary Movie 7 - Figure 4 Imaris asymmetric division event reconstruction. Displayed at 3 frames per second. Scale bar, 10 µm.

Supplementary Movie 8 - Maximum intensity projection of Spy650-DNA (white) labeled nuclei in StrataChip visualized via time-lapse light sheet microscopy showing nuclear degradation. Frames collected every 3 mins and displayed at 7 frames per second. Z-step, 0.23 nm/pixel. Scale bar, 10 µm.

Supplementary Table 1 – Differentially Expressed Genes (DEGs) between cell clusters

Supplementary Table 2 – Gene Ontology (GO) terms for cell clusters

Supplementary File 1 – Adobe Illustrator mask of StrataChip
